# 1-Methyl-3-phenyl­sulfonyl-2-piperidone

**DOI:** 10.1107/S1600536808009288

**Published:** 2008-04-16

**Authors:** Julio Zukerman-Schpector, Paulo R. Olivato, Carlos R. Cerqueira Jr, Elisângela Vinhato, Edward R. T. Tiekink

**Affiliations:** a Department of Chemistry, Universidade Federal de São Carlos, 13565-905 São Carlos, SP, Brazil; b Chemistry Institute, University of São Paulo, 05508-000 São Paulo, SP, Brazil; c Department of Chemistry, The University of Texas at San Antonio, One UTSA Circle, San Antonio, Texas 78249-0698, USA

## Abstract

The piperidone ring in the title compound, C_12_H_15_NO_3_S, has a slightly distorted half-chair conformation with the methyl, carbonyl and phenyl­sulfonyl ring substituents occupying equatorial, equatorial and axial positions, respectively. Mol­ecules are connected into centrosymmetric dimers *via* C—H⋯O inter­actions and these associate into layers *via* C—H⋯O—S contacts. Further C—H⋯O inter­actions involving both the carbonyl and sulfonyl O atoms consolidate the crystal packing by providing connections between the layers.

## Related literature

For related structures, see: Zukerman-Schpector *et al.* (1999 ▶, 2006 ▶). For related literature, see: Distefano *et al.* (1991 ▶); Olivato *et al.* (1992 ▶, 1997 ▶, 2003 ▶, 2004 ▶); Dal Colle *et al.* (1995 ▶). For ring conformational analysis, see: Cremer & Pople (1975 ▶). For the synthesis, see: Drabowicz *et al.* (1983 ▶); Zoretic & Soja (1976 ▶).
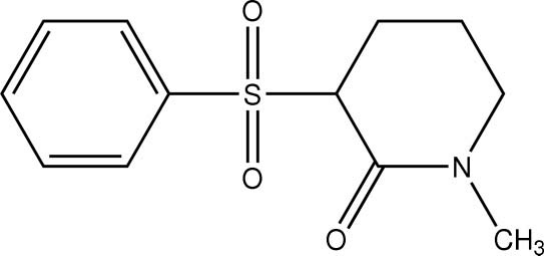


## Experimental

### 

#### Crystal data

C_12_H_15_NO_3_S
*M* *_r_* = 253.32
Monoclinic, 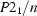

*a* = 9.0191 (16) Å
*b* = 10.4920 (18) Å
*c* = 13.446 (3) Å
β = 107.861 (3)°
*V* = 1211.1 (4) Å^3^
*Z* = 4
Mo *K*α radiation
μ = 0.26 mm^−1^
*T* = 98 (2) K
0.25 × 0.18 × 0.10 mm

#### Data collection

Rigaku AFC12κ/SATURN724 diffractometer
Absorption correction: multi-scan (*ABSCOR*; Higashi, 1995 ▶) *T*_min_ = 0.945, *T*_max_ = 0.974
5193 measured reflections
2729 independent reflections
2549 reflections with *I* > 2σ(*I*)
*R*_int_ = 0.025

#### Refinement

*R*[*F* ^2^ > 2σ(*F* ^2^)] = 0.047
*wR*(*F* ^2^) = 0.118
*S* = 1.12
2729 reflections
154 parameters
H-atom parameters constrained
Δρ_max_ = 0.39 e Å^−3^
Δρ_min_ = −0.45 e Å^−3^

### 

Data collection: *CrystalClear* (Rigaku, 2005 ▶); cell refinement: *CrystalClear*; data reduction: *CrystalClear*; program(s) used to solve structure: *SIR92* (Altomare *et al.*, 1999 ▶); program(s) used to refine structure: *SHELXL97* (Sheldrick, 2008 ▶); molecular graphics: *ORTEPII* (Johnson, 1976 ▶) and *DIAMOND* (Brandenburg, 2006 ▶); software used to prepare material for publication: *SHELXL97*.

## Supplementary Material

Crystal structure: contains datablocks global, I. DOI: 10.1107/S1600536808009288/ng2443sup1.cif

Structure factors: contains datablocks I. DOI: 10.1107/S1600536808009288/ng2443Isup2.hkl

Additional supplementary materials:  crystallographic information; 3D view; checkCIF report

## Figures and Tables

**Table 1:** Hydrogen-bond geometry (Å, °)

*D*—H⋯*A*	*D*—H	H⋯*A*	*D*⋯*A*	*D*—H⋯*A*
C2—H2⋯O1^i^	1.00	2.29	3.272 (2)	168
C6—H6*B*⋯O2^ii^	0.98	2.55	3.424 (3)	148
C11—H11⋯O3^iii^	0.95	2.62	3.224 (3)	122
C4—H4*A*⋯O1^iv^	0.99	2.48	3.328 (2)	144

Symmetry codes: (i) 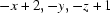; (ii) 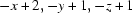; (iii) 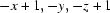; (iv) 
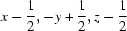
.

**Fig. 1.**
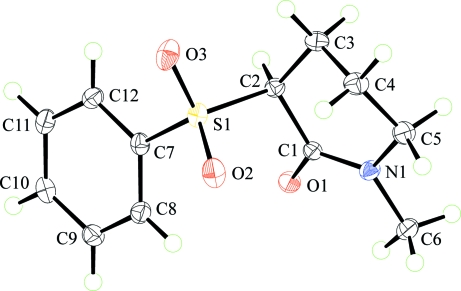
Molecular structure of (I) showing atom labelling and displacement ellipsoids at the 50% probability level.

**Fig. 2.**
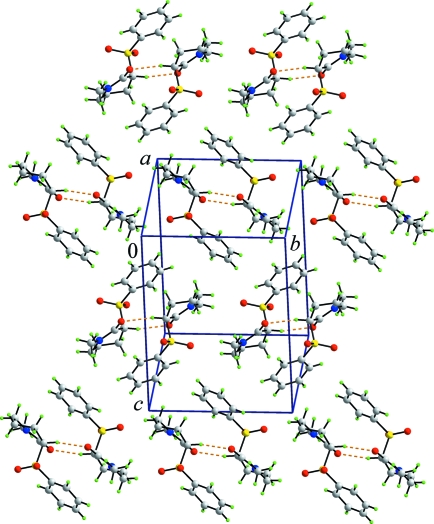
Crystal packing in (I) highlighting the C—H···O hydrogen bonding contacts (orange dashed lines) leading to the formation of dimeric aggregates and the overall layer arrangement.

## supplementary crystallographic information

### Comment

The title compound (I), Fig. 1, was studied as a part of an on-going investigation of conformational and electronic aspects of different classes of β-keto-sulfones, *i.e.*α-phenylsulfonyl -acetones, -acetophenones and -cyclohexanones, utilizing spectroscopic, theoretical and X-ray diffraction methods (Dal Colle *et al.*, 1995; Zukerman-Schpector *et al.*, 1999; 2006).

The piperidone ring has a slightly distorted half-chair conformation with a tendency towards a half-boat conformation: the ring-puckering parameters are q_2_ = 0.340 (2) Å, q_3_ = 0.332 (2) Å, QT = 0.476 (2) °, φ_2_ = -145.0 (3)° (Cremer & Pople, 1975). The ring substituents, *i.e. N*-methyl, *C*-carbonyl and *C*-phenylsulfonyl, occupy equatorial, equatorial and axial positions, respectively.

The crystal packing is dominated by C—H···O interactions, Table 1. Centrosymmetrically related molecules of (I) are connected into dimeric aggregates *via* C2—H···O1 contacts and these are linked into layers stacked along (1 0 1) *via* C6—H···O2 contacts. Connections betweem layers are also of the type C—H···O and serve to consolidate the crystal packing.

### Experimental

Initially, the 3-phenylsulfanyl-1-methyl-2-piperidone was obtained from the reaction of 1-methyl-2-piperidinone and diphenyl disulfide with LDA in THF as described in the literature (Zoretic and Soja, 1976). The product was oxidized with H_2_O_2_ and SeO_2_ (as catalyst) in methanol (Drabowicz *et al.* 1983) to give compound (I). After extraction with chloroform and subsequent evaporation, a crude solid was obtained. This product was subjected to flash chromatography with a solution of ethyl acetate and acetone in a 7:3 ratio. Suitable crystals were obtained by vapor diffusion from chloroform/n-hexane at 283 K.; m.p. 414–415 K. IR (cm^-1^): ν(C=O) 1652, ν(SO_2_)(as) 1307, ν(SO_2_)(*s*) 1148. NMR (CDCl_3_, p.p.m.): δ 1.79–2.74 (4*H*, m), 2.95 (3*H*, s), 3.30–3.48 (2*H*, m), 3.97 (1*H*, triplet, J = 6.1 Hz), 7.53–7.57 (2*H*, m, aryl-H), 7.62–7.67 (1*H*, m, aryl-H), 7.92–7.94 (2*H*, m, aryl-H). Analysis found: C 56.86, H 6.04, N 5.58; C_12_H_15_O_3_NS requires: C 56.89, H 5.97, N 5.53%.

### Refinement

All H atoms were included in the riding-model approximation with C—H = 0.95 - 1.00 Å, and with *U*_iso_(H) = 1.5*U*_eq_(methyl-C) or 1.2*U*_eq_(remaining-C).

### Crystal data

**Table:**

C_12_H_15_NO_3_S	*F*_000_ = 536
*M**_r_* = 253.32	*D*_x_ = 1.389 Mg m^−^^3^
Monoclinic, *P*2_1_/*n*	Mo *K*α radiation λ = 0.71070 Å
Hall symbol: -P 2yn	Cell parameters from 4417 reflections
*a* = 9.0191 (16) Å	θ = 2.4–40.6º
*b* = 10.4920 (18) Å	µ = 0.26 mm^−^^1^
*c* = 13.446 (3) Å	*T* = 98 (2) K
β = 107.861 (3)º	Block, colourless
*V* = 1211.1 (4) Å^3^	0.25 × 0.18 × 0.10 mm
*Z* = 4	

### Data collection

**Table:**

Rigaku AFC12κ/SATURN724 diffractometer	2729 independent reflections
Radiation source: fine-focus sealed tube	2549 reflections with *I* > 2σ(*I*)
Monochromator: graphite	*R*_int_ = 0.025
*T* = 98(2) K	θ_max_ = 27.5º
ω scans	θ_min_ = 2.5º
Absorption correction: multi-scan(ABSCOR; Higashi, 1995)	*h* = −10→11
*T*_min_ = 0.945, *T*_max_ = 0.974	*k* = −13→11
5193 measured reflections	*l* = −17→7

### Refinement

**Table:**

Refinement on *F*^2^	Secondary atom site location: difference Fourier map
Least-squares matrix: full	Hydrogen site location: inferred from neighbouring sites
*R*[*F*^2^ > 2σ(*F*^2^)] = 0.047	H-atom parameters constrained
*wR*(*F*^2^) = 0.118	*w* = 1/[σ^2^(*F*_o_^2^) + (0.0539*P*)^2^ + 0.7818*P*] where *P* = (*F*_o_^2^ + 2*F*_c_^2^)/3
*S* = 1.12	(Δ/σ)_max_ < 0.001
2729 reflections	Δρ_max_ = 0.39 e Å^−^^3^
154 parameters	Δρ_min_ = −0.45 e Å^−^^3^
Primary atom site location: structure-invariant direct methods	Extinction correction: none

### Special details

**Table:**

Geometry. All e.s.d.'s (except the e.s.d. in the dihedral angle between two l.s. planes) are estimated using the full covariance matrix. The cell e.s.d.'s are taken into account individually in the estimation of e.s.d.'s in distances, angles and torsion angles; correlations between e.s.d.'s in cell parameters are only used when they are defined by crystal symmetry. An approximate (isotropic) treatment of cell e.s.d.'s is used for estimating e.s.d.'s involving l.s. planes.
Refinement. Refinement of *F*^2^ against ALL reflections. The weighted *R*-factor *wR* and goodness of fit *S* are based on *F*^2^, conventional *R*-factors *R* are based on *F*, with *F* set to zero for negative *F*^2^. The threshold expression of *F*^2^ > σ(*F*^2^) is used only for calculating *R*-factors(gt) *etc*. and is not relevant to the choice of reflections for refinement. *R*-factors based on *F*^2^ are statistically about twice as large as those based on *F*, and *R*- factors based on ALL data will be even larger.

### Fractional atomic coordinates and isotropic or equivalent isotropic displacement parameters (Å^2^)

**Table:**

	*x*	*y*	*z*	*U*_iso_*/*U*_eq_	
S1	0.76305 (5)	0.20597 (4)	0.49677 (3)	0.01768 (15)	
O1	1.13033 (15)	0.14856 (13)	0.56636 (9)	0.0186 (3)	
O2	0.79732 (18)	0.33520 (13)	0.53443 (11)	0.0265 (3)	
O3	0.60745 (15)	0.17739 (15)	0.43018 (11)	0.0275 (3)	
N1	1.11807 (17)	0.30781 (15)	0.45077 (11)	0.0170 (3)	
C1	1.0585 (2)	0.20646 (17)	0.48622 (13)	0.0147 (3)	
C2	0.8952 (2)	0.16205 (17)	0.42479 (13)	0.0153 (3)	
H2	0.8969	0.0669	0.4210	0.018*	
C3	0.8348 (2)	0.21313 (18)	0.31267 (14)	0.0196 (4)	
H3A	0.8843	0.1657	0.2676	0.024*	
H3B	0.7208	0.1998	0.2850	0.024*	
C4	0.8711 (2)	0.35463 (19)	0.30992 (14)	0.0214 (4)	
H4A	0.8313	0.3865	0.2372	0.026*	
H4B	0.8188	0.4027	0.3530	0.026*	
C5	1.0457 (2)	0.37553 (19)	0.35160 (14)	0.0208 (4)	
H5A	1.0942	0.3460	0.2989	0.025*	
H5B	1.0668	0.4679	0.3625	0.025*	
C6	1.2777 (2)	0.3460 (2)	0.50751 (14)	0.0213 (4)	
H6A	1.3156	0.2956	0.5717	0.032*	
H6B	1.2794	0.4367	0.5254	0.032*	
H6C	1.3448	0.3314	0.4635	0.032*	
C7	0.8027 (2)	0.10460 (17)	0.60682 (13)	0.0166 (3)	
C8	0.9117 (2)	0.14160 (19)	0.70016 (14)	0.0199 (4)	
H8	0.9681	0.2189	0.7041	0.024*	
C9	0.9368 (2)	0.0641 (2)	0.78751 (14)	0.0211 (4)	
H9	1.0114	0.0879	0.8516	0.025*	
C10	0.8528 (2)	−0.04843 (19)	0.78116 (14)	0.0215 (4)	
H10	0.8704	−0.1013	0.8411	0.026*	
C11	0.7431 (2)	−0.08410 (19)	0.68753 (15)	0.0209 (4)	
H11	0.6856	−0.1607	0.6839	0.025*	
C12	0.7177 (2)	−0.00801 (18)	0.59943 (14)	0.0185 (4)	
H12	0.6437	−0.0322	0.5352	0.022*	

### Atomic displacement parameters (Å^2^)

**Table:**

	*U*^11^	*U*^22^	*U*^33^	*U*^12^	*U*^13^	*U*^23^
S1	0.0168 (2)	0.0151 (3)	0.0225 (2)	0.00200 (16)	0.00796 (18)	0.00263 (16)
O1	0.0179 (6)	0.0205 (7)	0.0155 (6)	0.0007 (5)	0.0026 (5)	0.0026 (5)
O2	0.0382 (8)	0.0134 (7)	0.0353 (8)	0.0021 (6)	0.0223 (7)	0.0004 (6)
O3	0.0144 (6)	0.0339 (8)	0.0324 (7)	0.0022 (6)	0.0044 (6)	0.0120 (6)
N1	0.0153 (7)	0.0180 (8)	0.0159 (7)	−0.0018 (6)	0.0023 (6)	0.0013 (6)
C1	0.0153 (8)	0.0149 (9)	0.0147 (7)	−0.0002 (6)	0.0056 (6)	−0.0023 (6)
C2	0.0153 (8)	0.0141 (8)	0.0163 (8)	0.0000 (6)	0.0046 (6)	−0.0010 (6)
C3	0.0181 (9)	0.0224 (10)	0.0154 (8)	0.0001 (7)	0.0008 (7)	−0.0005 (7)
C4	0.0218 (9)	0.0215 (9)	0.0178 (8)	0.0016 (7)	0.0015 (7)	0.0027 (7)
C5	0.0242 (9)	0.0193 (9)	0.0174 (8)	−0.0015 (7)	0.0043 (7)	0.0044 (7)
C6	0.0187 (9)	0.0233 (10)	0.0205 (8)	−0.0054 (7)	0.0039 (7)	0.0003 (7)
C7	0.0180 (8)	0.0145 (8)	0.0194 (8)	0.0015 (7)	0.0090 (7)	0.0004 (7)
C8	0.0192 (9)	0.0192 (9)	0.0232 (9)	−0.0026 (7)	0.0096 (7)	−0.0035 (7)
C9	0.0197 (9)	0.0254 (10)	0.0191 (8)	−0.0010 (7)	0.0072 (7)	−0.0037 (7)
C10	0.0230 (9)	0.0243 (10)	0.0205 (8)	0.0027 (8)	0.0113 (7)	0.0037 (7)
C11	0.0204 (9)	0.0180 (9)	0.0274 (9)	−0.0019 (7)	0.0120 (7)	0.0002 (7)
C12	0.0173 (8)	0.0170 (9)	0.0214 (8)	−0.0014 (7)	0.0061 (7)	−0.0018 (7)

### Geometric parameters (Å, °)

**Table:**

S1—O3	1.4457 (15)	C5—H5A	0.9900
S1—O2	1.4472 (15)	C5—H5B	0.9900
S1—C7	1.7674 (18)	C6—H6A	0.9800
S1—C2	1.8101 (18)	C6—H6B	0.9800
O1—C1	1.233 (2)	C6—H6C	0.9800
N1—C1	1.343 (2)	C7—C8	1.391 (3)
N1—C6	1.463 (2)	C7—C12	1.395 (3)
N1—C5	1.475 (2)	C8—C9	1.389 (3)
C1—C2	1.524 (2)	C8—H8	0.9500
C2—C3	1.534 (2)	C9—C10	1.391 (3)
C2—H2	1.0000	C9—H9	0.9500
C3—C4	1.523 (3)	C10—C11	1.393 (3)
C3—H3A	0.9900	C10—H10	0.9500
C3—H3B	0.9900	C11—C12	1.388 (3)
C4—C5	1.517 (3)	C11—H11	0.9500
C4—H4A	0.9900	C12—H12	0.9500
C4—H4B	0.9900		
			
O3—S1—O2	118.26 (9)	N1—C5—C4	112.68 (15)
O3—S1—C7	107.61 (9)	N1—C5—H5A	109.1
O2—S1—C7	107.71 (9)	C4—C5—H5A	109.1
O3—S1—C2	106.81 (9)	N1—C5—H5B	109.1
O2—S1—C2	108.70 (8)	C4—C5—H5B	109.1
C7—S1—C2	107.28 (8)	H5A—C5—H5B	107.8
C1—N1—C6	117.93 (15)	N1—C6—H6A	109.5
C1—N1—C5	126.04 (15)	N1—C6—H6B	109.5
C6—N1—C5	115.53 (15)	H6A—C6—H6B	109.5
O1—C1—N1	122.70 (16)	N1—C6—H6C	109.5
O1—C1—C2	118.95 (16)	H6A—C6—H6C	109.5
N1—C1—C2	118.35 (15)	H6B—C6—H6C	109.5
C1—C2—C3	114.75 (15)	C8—C7—C12	121.42 (17)
C1—C2—S1	108.53 (11)	C8—C7—S1	119.54 (14)
C3—C2—S1	110.06 (12)	C12—C7—S1	118.96 (14)
C1—C2—H2	107.8	C9—C8—C7	119.10 (18)
C3—C2—H2	107.8	C9—C8—H8	120.4
S1—C2—H2	107.8	C7—C8—H8	120.4
C4—C3—C2	110.50 (15)	C8—C9—C10	120.03 (17)
C4—C3—H3A	109.6	C8—C9—H9	120.0
C2—C3—H3A	109.6	C10—C9—H9	120.0
C4—C3—H3B	109.6	C9—C10—C11	120.40 (17)
C2—C3—H3B	109.6	C9—C10—H10	119.8
H3A—C3—H3B	108.1	C11—C10—H10	119.8
C5—C4—C3	109.79 (16)	C12—C11—C10	120.17 (18)
C5—C4—H4A	109.7	C12—C11—H11	119.9
C3—C4—H4A	109.7	C10—C11—H11	119.9
C5—C4—H4B	109.7	C11—C12—C7	118.87 (17)
C3—C4—H4B	109.7	C11—C12—H12	120.6
H4A—C4—H4B	108.2	C7—C12—H12	120.6
			
C6—N1—C1—O1	3.1 (3)	C1—N1—C5—C4	21.7 (3)
C5—N1—C1—O1	174.61 (17)	C6—N1—C5—C4	−166.58 (16)
C6—N1—C1—C2	−177.19 (15)	C3—C4—C5—N1	−47.8 (2)
C5—N1—C1—C2	−5.7 (3)	O3—S1—C7—C8	−154.80 (15)
O1—C1—C2—C3	−163.27 (15)	O2—S1—C7—C8	−26.27 (17)
N1—C1—C2—C3	17.0 (2)	C2—S1—C7—C8	90.58 (15)
O1—C1—C2—S1	73.16 (18)	O3—S1—C7—C12	21.86 (17)
N1—C1—C2—S1	−106.55 (15)	O2—S1—C7—C12	150.39 (14)
O3—S1—C2—C1	172.73 (12)	C2—S1—C7—C12	−92.76 (15)
O2—S1—C2—C1	44.09 (14)	C12—C7—C8—C9	0.5 (3)
C7—S1—C2—C1	−72.12 (14)	S1—C7—C8—C9	177.05 (14)
O3—S1—C2—C3	46.40 (15)	C7—C8—C9—C10	−0.5 (3)
O2—S1—C2—C3	−82.24 (14)	C8—C9—C10—C11	0.0 (3)
C7—S1—C2—C3	161.55 (12)	C9—C10—C11—C12	0.5 (3)
C1—C2—C3—C4	−44.1 (2)	C10—C11—C12—C7	−0.5 (3)
S1—C2—C3—C4	78.63 (17)	C8—C7—C12—C11	0.0 (3)
C2—C3—C4—C5	59.6 (2)	S1—C7—C12—C11	−176.58 (14)

### Hydrogen-bond geometry (Å, °)

**Table:**

*D*—H···*A*	*D*—H	H···*A*	*D*···*A*	*D*—H···*A*
C2—H2···O1^i^	1.00	2.29	3.272 (2)	168
C6—H6B···O2^ii^	0.98	2.55	3.424 (3)	148
C11—H11···O3^iii^	0.95	2.62	3.224 (3)	122
C4—H4A···O1^iv^	0.99	2.48	3.328 (2)	144

Symmetry codes: (i) −*x*+2, −*y*, −*z*+1; (ii) −*x*+2, −*y*+1, −*z*+1; (iii) −*x*+1, −*y*, −*z*+1; (iv) *x*−1/2, −*y*+1/2, *z*−1/2.
